# 1407. Migration Timing and its Association with Disease Features in a Chagas Disease Cohort in New England

**DOI:** 10.1093/ofid/ofad500.1244

**Published:** 2023-11-27

**Authors:** Alyse Wheelock, Katherine Reifler, Davidson H Hamer, Natasha Hochberg, Deepa Gopal, Daniel Bourque

**Affiliations:** Boston University School of Medicine, Boston, Massachusetts; Boston University School of Medicine, Boston, Massachusetts; Boston University School of Public Health, MA; Boston Medical Center, Boston, Massachusetts; Boston University School of Medicine, Boston, Massachusetts; Boston Medical Center / Boston University School of Medicine, Boston, Massachusetts

## Abstract

**Background:**

Chagas disease is under-recognized in the United States (US) and little is known about how time spent living in endemic versus non-endemic settings affects features of the disease.

**Methods:**

We performed a cross-sectional, retrospective analysis of 108 individuals with Chagas disease. Presence of cardiac disease was defined according to the American Heart Association classification of Chagas cardiomyopathy. We performed a multivariate logistic regression adjusting for country of birth (COB, analyzed as El Salvador vs. other) and age at confirmatory diagnosis to evaluate whether proportion of lifetime in COB was associated with cardiac disease. We also performed an exploratory analysis of semi-quantitative *Trypanosoma cruzi* ELISA optical density (OD) values to assess for association with proportion of lifetime in COB, including the same covariates in a multivariate linear regression.

**Results:**

This patient cohort was predominantly from El Salvador (92/108, 85%) and other countries in Central America (11/108, 10%), with a median age at diagnosis of 44 years (interquartile range [IQR] 18). Most individuals originated from rural areas (84/90, 93%) and immigrated to the US in adulthood (median 26 years, IQR 13) after having lived a median of 70% (IQR 25) of their life in their COB. Age at migration and age at confirmatory diagnosis were highly correlated (Pearson’s correlation coefficient [CC]= 0.69, p< 0.001). Confirmatory diagnosis occurred a median of 13 years (IQR 14) after arrival in the US. Cardiac disease was present in 47/108 (44%). Adjusting for age at diagnosis and COB, the proportion of lifetime in COB was not significantly associated with cardiac disease (odds ratio 5.6, [95% CI 0.355 - 87.1], p=0.22), nor was it associated with higher OD values (CC = –0.2, 95% CI –1.2-0.9, p=0.74).

Cohort demographics and time to Chagas disease diagnosis after arrival in the U.S.

**Figure:**
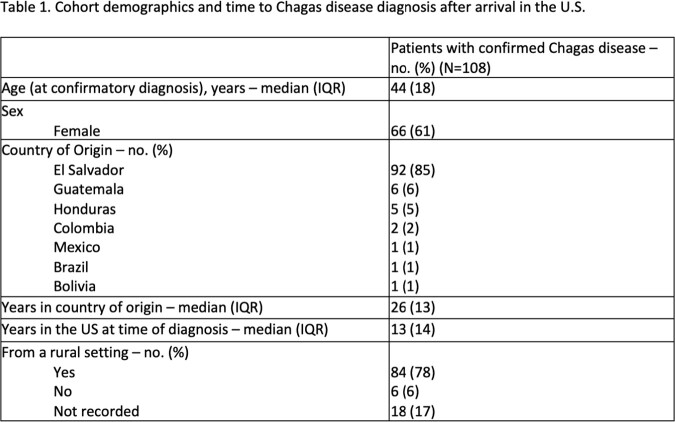

Features of migration history and Chagas cardiac disease status

**Figure:**
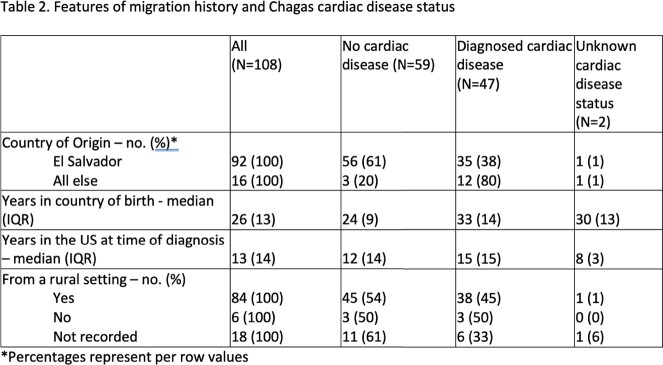

**Conclusion:**

In this cohort of individuals with Chagas disease, there was a substantial delay in diagnosis after arrival to the US, which may have been longer if not for local screening efforts. Cardiac disease was prevalent but not associated with higher proportion of time spent in COB. Exploratory analysis of serology titers was not associated with proportion of lifetime spent in COB, contrary to our expectation.

**Disclosures:**

**Davidson H. Hamer, MD**, Kephera Diagnostics: Grant/Research Support|Takeda: Advisor/Consultant|Takeda: Grant/Research Support|Trinity Biotech, LLC: Advisor/Consultant|Valneva: Advisor/Consultant|Valneva: Grant/Research Support **Natasha Hochberg, MD, MPH**, Novartis Institute for Biomedical Research: Full-time employment|Novartis Institute for Biomedical Research: Stocks/Bonds **Daniel Bourque, MD**, Kephera Diagnostics: Grant/Research Support

